# Relationship Between Direct Aggression and Prosocial Behavior: The Role of Attention and Intelligence Among Children at Risk for Behavioral Problems

**DOI:** 10.1007/s10578-024-01738-7

**Published:** 2024-08-16

**Authors:** Sofia Lira Chiodi, Patrícia Silva Lúcio, Beatriz Ilari, Nayana Di Giuseppe Germano, Hugo Cogo-Moreira, Graziela Bortz

**Affiliations:** 1 Graduate Program in Psychology, State University of Londrina, Londrina, Brazil; 2 Department of Psychology and Psychoanalysis, State University of Londrina, Rodovia Celso Garcia Cid, PR 445, Km 380, Campus Universitário, 86057970, Londrina, Paraná Brazil; 3 Graduate Program of Pshychology of Development and Learning, São Paulo State University, Bauru, Brazil; 4 Department of Music Teaching and Learning, Thornton School of Music, University of Southern California, Los Angeles, CA USA; 5 Department of Music, Federal University of Santa Maria, Rio Grande Do Sul, Brazil; 6 Department of Education, ICT and Learning, Østfold University College, Halden, Norway; 7 Music Department, Arts Institute of Unesp, São Paulo State University, São Paulo, Brazil

**Keywords:** Direct aggression, Prosocial behavior, Attention, Intelligence, Moderation

## Abstract

This study aimed to investigate the influence of attention and intelligence in the prediction of prosocial behavior by direct aggression (proactive or reactive) in school-aged children at risk for behavioral problems. The sample was composed of 64 children aged 6 to 8 years screened for risk of behavioral problems, who were enrolled in a clinical trial. Multiple regression models were tested to investigate the prediction of prosocial behavior by direct aggression (proactive or reactive), attention, and intelligence. Additive multiple moderation models were tested to analyze the conditional effect of attention and intelligence in the prediction of prosocial behavior by proactive and reactive aggression. Aggression (proactive or reactive), attention, and intelligence did not linearly predict prosocial behavior. Conditional effects were found only for the proactive aggression model. Negative impacts on prosocial behavior were observed among children with low attention and high intelligence performance, while medium and high levels of attention showed to be protective factors among low to medium intellectual ability children. Clinical impacts of the results are discussed.

**Supplementary Information:**

The online version contains supplementary material available at 10.1007/s10578-024-01738-7.

## Introduction

Aggression and prosocial behavior are different means of responding to the social environment and can be used for solving social problems. Historically, both behaviors were set in opposite sides of social behavior patterns [1]. Prosocial behavior is defined as a set of social behaviors that may benefit others, which encompasses the other-oriented and independent abilities of helping, comforting, and sharing [2]. Some types of these behaviors are presented by toddlers from the second year of life [3–7], and increase in the following years, when the ability of differentiating self and others improves [8, 9]. Prosocial behavior is multidetermined, and different factors can influence its development such as genetics, neurophysiological processes, and family (e.g., parental styles practices and emotional socialization). The same is valid about its manifestation, varying, for example, according to context, function, socio-cognitive processes including perspective-taking skills and moral reasoning, and socialization with teachers and peers [8].

Aggression, in turn, is characterized by physical and verbal (direct aggression) or social behaviors (indirect aggression, e.g., exclusion of a group or gossip) that can hurt or even harm another person. Behaviors characterized as aggression emerge early in development, and its trajectory is heterogeneous. Generally, physical aggression such as hitting, biting, and kicking, is reported in the first year of life [10–14]. Its prevalence normally stabilizes or increases in toddlerhood [12, 15, 16], and decreases in childhood and adolescence, owing to the enhancement of cognitive abilities, emotional regulation, language skills, and socialization [17–20]. In childhood and adolescence, other types of aggression emerge such as verbal and indirect aggression [21, 22]. Nevertheless, this is a period when manifestation of prosocial behavior increases [23, 24].

The relation between aggression and prosocial behavior may be different when the function of aggression is considered. Direct aggression behaviors (i.e., overt aggression such as physical and verbal behaviors) can be goal-oriented (i.e., proactive aggression) or a reaction to others’ aggression (i.e., reactive aggression). Direct aggressions are related to maladjustment outcomes in childhood and adolescence, as emotional dysregulation, conduct problems, low peer acceptance, peer rejection, and deficits in prosocial behavior (for a metanalysis, see Card et al. [25]). Therefore, prosocial behaviors may function as protective factors for some of these outcomes, since they can be used to reduce the harmful effects of aggression, reduce sanctions, and reactions [23, 26].

Scholars tend to study both of these social behaviors separately and independently [27–31], with intervention studies being often designed to improve prosociality and decrease aggression [32–37]. Nevertheless, there is no consistent evidence to support that these two behaviors are on opposite ends of a spectrum.

Although in most studies measures of direct aggression are typically defined in relationship to proactive or reactive functions, studies have shown no associations [38–40] or a weak to moderate negative association between proactive and reactive aggression and prosocial behaviors in school-aged children [23, 29, 41–46], including a metanalysis [25]. The joint development of direct aggression and prosocial behavior follows a heterogeneous trajectory, with several studies reporting their co-occurrence at different levels (e.g., [9, 23, 24, 40]). In general, these results suggest that aggression and prosociality are not opposite categories nor extremes in a same spectrum, and that children could exhibit both behaviors at low, moderate, or high levels.

Even if the onset of aggression emerges prior to prosocial behavior during child development, little attention has been given to the impact of aggression on the expression of prosocial behavior. At the time of writing, we found only one study that investigated the prediction of prosocial behavior by direct aggression [43]. This longitudinal investigation with 1334 Swiss children aged 7–11 demonstrated that the manifestation of aggression predicts prosocial behavior over time, while the reverse does not happen. The authors concluded that aggression leads to peers’ relationship difficulties and, consequently, to fewer chances of developing and improving prosocial behavior. Therefore, one of the aims of the present study is to explore the prediction of prosocial behavior by direct aggression (proactive and reactive) in children at risk for behavior problems.

In addition to behavioral influences, the development and manifestation of aggression and prosocial behaviors can be affected by cognition (e.g., [14, 47–50]). To behave and react properly in a social environment, the child must be cognitively able to adequately encode, perceive, and interpret the available social cues and decide how to solve problems that arise in the context of social interactions. Social information-processing is a multiple-step model useful for explaining the cognitive mechanisms involved in aggression through a social problem-solving situation [51]. These sequential steps comprise the perception and decoding of social cues, decision making of possible behavioral responses, and enacting. Children do so by engaging cognitive processes involved in attention [52] and fluid intelligence [53]. For example, aggression has been linked to attentional deficits and biases in social information processing [49]. Therefore, attention and fluid intelligence (i.e., problem solving) appear to impact the expression of social behaviors.

Research relating aggression and prosocial behaviors to mechanisms of attention and intelligence tend to involve their social or emotional counterparts (e.g., [54–58]). Little is known about the relationship between these social behaviors to the cognitive facets of attention and intelligence. In general, these studies demonstrate that deficits in prosocial behavior and moderate/high levels of aggression are related to lower levels of intelligence or attention [14, 59, 60]. Therefore, this work explores the relationship between aggression, prosocial behavior, attention and fluid intelligence (i.e., problem solving).

Moreover, according to Cochrane et al. [61], there are few studies investigating direct correlations between attention and fluid intelligence, including measures of attention control. For a series of visual attentional measures, the authors found positive, low to moderate, correlations with the Raven’s test for a sample of children. The authors also demonstrated that working memory mediates the relationship between visual attention and fluid intelligence, but not the opposite. Consequently, an interaction between fluid intelligence and visual attention may be not expected. Considering the social information processing model in which attentional and problem-solving processes is involved, therefore, if the relationship between aggression and prosocial behavior is conditioned by attention and intelligence, an additional multiple moderation model will be better suited than a moderated moderation model [62].

To address the relationship between prosocial behavior and direct aggression, attention and intelligence, the present study had two aims. First, we aimed to test the predictive contribution of peers’ direct aggression (proactive or reactive), attention, and intelligence to prosocial behavior in children at risk for behavioral problems. Second, we explored the conditional effect (i.e., interaction) of the cognitive processes of intelligence and attention in the prediction of prosocial behaviors by direct aggression (proactive and reactive) in children at risk for behavioral problems. In other words, we asked: Is the impact of direct aggression on prosocial behavior affected by attention or intelligence among this group of children?

As previously noted, according to the few studies available, aggression, attention, and intelligence are not strong predictors of prosocial behavior. Nevertheless, we hypothesize that attentional or problem-solving deficits can impair one or more steps of social information processing. For example, the child may not perceive and accurately decode a social cue, disturbing his/her interpretation or decision-making/problem-solving process. Alternatively, and despite perceiving and decoding accurately the social cue, the child may not have sufficient skills to support their decision making and problem solving. Thereby, although a low to negligible prediction may be expected from aggression, intelligence, and attention on prosocial behavior, these variables may interact, generating a new relationship. Hence, this study explored attention and intelligence as candidates to moderate the relationship between aggression and prosocial behaviors.

## Method

### Design and Ethics

Data for the present study came from a quasi-experimental study which aimed to examine the impact of a musical intervention against treatment as usual (control group under no intervention) in the behavior, cognitive abilities, and brain structures of children who are at risk for mental health problems (Universal Trial Number: U1111-1243-8071). This study was approved by the Ethical Committee for Research Involving Human Beings of the Universidade Estadual Paulista Julio de Mesquita Filho (São Paulo, Brazil) and is in accordance with the Declaration of Helsinki. Parents signed consent forms and children gave their assent prior to participating in this study.

### Recruitment and Sample

Children in the experimental group were recruited through a partnership with the “Guri Santa Marcelina”, a music education and sociocultural inclusion program for children in vulnerable situation run by the Government of the State of São Paulo. The program covers areas of the city with high levels of poverty, urban violence, and risk exposure, which means that children are commonly exposed to vulnerable situations. The Guri Program has 34 centers (known as pólos) in the city of São Paulo and 10 centers in the metropolitan area that serve more than 600 children every year. For logistic reasons, centers in the metropolitan area were excluded for sample recruitment. From the 34 remaining centers, two were in the downtown area, five in the North, 14 in the South, and 13 in the East regions of São Paulo. The program does not have centers in the West region, which is a high-income area. Considering the population of São Paulo of almost 11.000,000 inhabitants [63], and its heterogeneous distribution, we sorted one center in the downtown area, two in the North and South, and six from the East regions, totaling 11 centers from which the experimental group was drawn.^1^

The Guri Program is an afterschool program, therefore the schedule for the music classes overlaps with the Brazilian academic calendar. About two weeks prior to the beginning of the 2020 school year, registration took place at the Guri Program site. During that time, a series of recruitment meetings were held, when we invited parents of children aged between 6 and 8 years, who were about to start first and second grades. Only children without a formal diagnostic of pervasive developmental disorders such as ASD or intellectual disability were eligible to take part in the study.

Once the goals of the study were fully explained and all questions answered, parents/legal guardians were invited to sign the informed consent form. Parents/legal guardians completed the Brazilian version of the Strengths and Difficulties Questionnaire (SDQ; [64, 65]) about their children. Those who met the inclusion criteria (i.e., achieving 10 or more points on the general difficulty scale) were invited to complete a battery of tests.^2^ Trained psychologists tested children individually, according to instructions given in the manuals, in a quiet room in their center. The tasks included a general intelligence scale, cognitive attention (sustained, divided, alternating forms), working memory, and a proactive and reactive aggression. Data from children whose IQ were below the standard score of 80 were not included in the analysis.

Following the composition of the experimental group, we compiled a list of schools attended by our participants and secured their permissions to recruit children to compose the control group. Teachers sent out the informed consent and the parents’ version of the SDQ through the students. Children whose parents signed the term and who reached the cutoffs for inclusion were invited to take part in the study.

Unfortunately, COVID-19 was declared a pandemic at the time of data collection, disrupting our study. By that time, we had 46 children of the experimental group and 18 children from the control group.^3^ Therefore, in the present study we aggregated data from baseline testing for the control and experimental groups for data analysis (*n* = 64). These groups did not differ because they came from the same school community and the musical classes had not started. Online Resource 1 presents the demographic characteristics of the final sample.

### Measures

#### Aggression

Q-Carp (Peer Aggressive and Reactive Behaviors Questionnaire) is a questionnaire that evaluates direct aggression and reaction to aggression by children in the school environment. It is the only questionnaire that assesses direct aggression with validation studies for the Brazilian’s children population. The questionnaire is a self-rated scale divided into two scales: Peer Aggression Scale (PAS) and Reaction to Peer Aggression Scale (RPA) [68]. The PAS scale is a measure of proactive aggression composed by 8 items, which evaluates children’s verbal or physical proactive aggression (e.g., How often do you kick or slap a classmate?). The response is given in a 4-point Likert scale, which varies from every day (3) to never (0). The total score is the sum of the 1, 3, 5, 6 and 8 items, and ranges from 0 to 15 (items 2, 4, and 7 are social-desirability bias items and are not considered for scoring).

The Reaction to Peer Aggression Scale (RPA) is a 12-item scale, which is divided in three subscales that assess strategies against peer aggression. The strategies are reactive aggression (6 items, e.g., “When a classmate makes fun of you, do you yell at him/her?”), seeking teacher support (3 items, e.g., “When a classmate breaks something of yours, do you tell the teacher?”), and internalizing reaction (3 items, e.g., “When a classmate makes fun of you, do you cry or pout?”). For this research, we used the scores of the reactive aggression subscale (RA). Rating is in a 4-point Likert scale format, which varies from always (3) to never (0). Therefore, the score of the reaction aggression subscale varies from 0 to 18.

#### Fluid Intelligence

The Brazilian version of the Raven’s Colored Progressive Matrices was used for assessing intelligence [69]. The test is composed by 36 items equally divided into three sets: A, Ab, and B. The child must choose the missing part that correctly complete the pattern of a figure among six response options. The total score is the sum of the correct answers (maximum score = 36).

#### Behavioral Problems Risk and Prosocial Behavior

Prosocial behavior was assessed using the prosocial behavior subscale of the Strengths and Difficulties Questionnaire (SDQ), a scale which screens behavioral problems in children aged 2 to 17 years [64]. The SDQ is composed by 25 items divided into five subscales: emotional symptoms (e.g., “Many worries or often seems worried”), conduct problems (e.g., “Often fights with other children or bullies them”), hyperactivity/inattention (e.g., “Restless, overactive, cannot stay still for long”), peer relationship problems (e.g., “Gets along better with adults than with other children”), and prosocial behavior (e.g., “Considerate of other people's feelings”). Each subscale has 5-item Likert-type scale with three points (0 = Not true, 1 = Somewhat true, and 2 = Certainly true), that correspond to the respondent’s level of agreement with each statement. Some items are reversed before scoring (e.g., “Thinks things out before acting”). The subscales score ranges from 0 to 10, and the higher the score, the greater the child's difficulties or the higher the prosocial behavior. Furthermore, the questionnaire has a general difficulty score generated by the sum of the subscales: emotional symptoms, conduct problems, hyperactivity/inattention, and peer relationship problems. For the present study, a cutoff of 10 points was used as an index of risk for difficulty problems. The SDQ can be rated by parents, teachers or self-rated, but in this study, we used the parents’ version for methodological issues. This allowed us to use the same instrument for both the control and experimental groups, as the teacher’s version could not be applied to the latter. For the present research, we used the validated Brazilian version of the scale [70, 71].

#### Sustained Attention

Attention was evaluated using the subtest of sustained attention from the *Bateria Psicológica para Avaliação da Atenção* (BPA; Psychological Battery for Attention Assessment), a test that assesses general cognitive attention [72]. The sustained attention task presents 120 visual target stimuli among 400 stimuli (in a 20 lines × 20 stimulus table). The individual must respond within two minutes. The score is the difference between the accuracy and the sum of errors and omissions, so that scores can achieve negative and positive values. For this study, we used total score in the task (maximum 108).

#### Working Memory

The digit span backward subtest from Wechsler Intelligence Scale—IV (Brazilian version) was used as a measure of working memory [73]. This task consists of 8 items presented in increasing order of difficulty. In each item, the evaluator reads a sequence of digits to the child, and, in turn, the child must repeat this sequence in the reverse order in which it was presented. In each item the child has two attempts, and for each attempt the following score is assigned: 0 points for errors and 1 for correct answers. The task presents two different scores: a total score (ranging from 0 to 16 points) and the span score, or the longest sequence numbers in memory kept by the child. For this research, we used only the span score. This measure was used due to its recognizable role in the evaluation of working memory (e.g., [74]).

### Statistical Analysis

Descriptive and inferential statistical analyses were performed to data analysis. To investigate our first aim, two models of multiple regression analysis were tested to analyze the prediction of prosocial behavior by proactive (Model 1) or reactive aggression (Model 2). The Enter Method was used in models modeling.

**Fig. 1:**
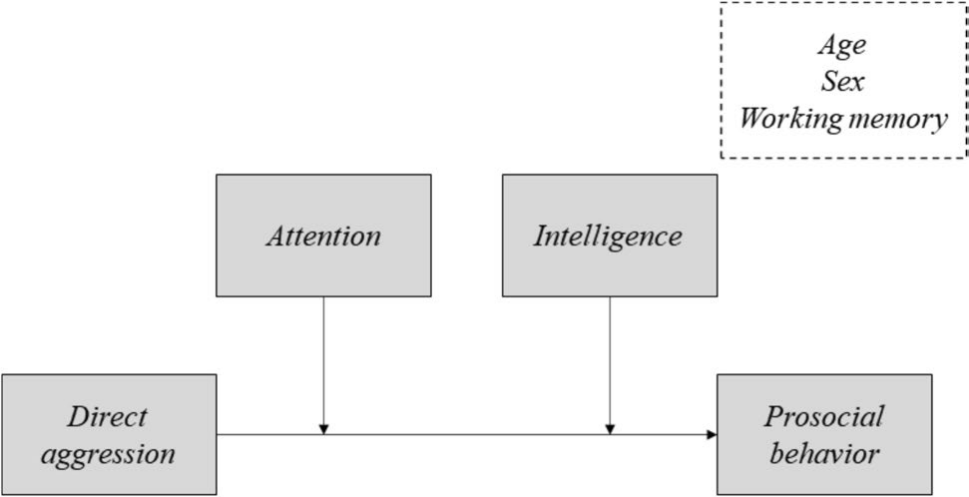
Conceptual model of the additive multiple moderation [62]. *Note* Direct aggression: In Model 1, direct aggression is measured by proactive aggression and in Model 2, by reactive aggression. Age, sex and working memory are covariates in the models

For our second aim, we tested additive multiple moderation models (Model 2, [62]), which included both attention and intelligence as moderators in the prediction of prosocial behavior by aggression (proactive or reactive). In another words, two hypothetical models were tested, one for each antecedent variable: (a) proactive aggression, and (b) reactive aggression. The outcome variable was the prosocial behavior, and the moderators were attention and intelligence. Figure 1 depicts the conceptual model of the multiple moderation models tested.

To test the conditional effect, attention and intelligence were treated as continuous variables, and it was used the values of the 16th, 50th and 84th percentiles (arbitrary sample values, automatically given by the program), classified as low, medium, and high performance, respectively. The attention values used were − 43, 29, and 43, and the fluid intelligence values were 14, 19, and 23 (all the values were calculated by the program).

A macro implementation of process (version 4.0) for SPSS (version 23.0) was used to data analysis [62]. Bootstrapping with 5000 bootstrap samples for confidence intervals of 95% (C. I. 95%) were generated, and the intervals that did not include zero were considered significant. The variables sex (reference variable was female), age, and working memory were included in the moderation models as covariates.

## Results

### Descriptive Analyzes

As overt peer aggression commonly differs between sexes (e.g., [19, 75]), a *t*-test was conducted using this variable. There were no significant differences in proactive aggression (*t*(62) = 0.379, *p* = 0.706) or reactive aggression (*t*(62) = 0.931, *p* = 0.355) between boys and girls.

Children’s difficulty scores ranged from 10 to 30 (mean = 17.5; SD = 4.18). Table 1 presents the descriptive statistics for the measures. Regarding the behaviors measured by the scales, children scores were at higher levels for reactive aggression (50% percentile 84th), were at middle of the distribution for proactive aggression (64.3% at percentile 50th) and were almost evenly spread at the prosocial behavior. Most of the children showed medium levels of intelligence and attention (respectively, 57.8% and 64.1% at percentile 50th), with the latter presenting fewer subjects at the extreme of the distribution. Only two correlations were found to be statistically significant. There were positive correlations between proactive and reactive aggression (*ρ* = 0.332; *p* = 0.007), and between sustained attention and fluid intelligence (*ρ* = 0.318; *p* = 0.010). Both correlations were weak in magnitude (Table 1).

**Table 1:** Descriptive statistics of the variables of the study

Variables		Mean	SD	Min	Max	Percentiles		
						16 (%)	50 (%)	84 (%)
1	Proactive aggression	12.53	3.09	2	15	18.8	43.8	37.5
2	Reactive aggression	15.11	4.36	0	18	15.6	34.4	50.0
3	Prosocial behavior	8.42	1.90	0	10	26.6	34.4	39.1
4	Sustained attention	11.42	38.60	− 80	64	17.2	64.1	18.8
5	Fluid intelligence	19.03	4.57	11	29	20.3	57.8	21.9

			Correlation					
			1	2	3	4	5	
1	Proactive aggression		–	**0.007**	0.352	0.591	0.188	
2	Reactive aggression		**0.332**	–	0.893	0.624	0.787	
3	Prosocial behavior		0.118	− 0.017	–	0.210	0.439	
4	Sustained attention		− 0.068	− 0.062	0.159	–	**0.010**	
5	Fluid Intelligence		− 0.167	− 0.034	− 0.098	**0.318**	–	

For correlations, in the bottom of the diagonal are the Pearson’s values and in the top the significance index. In bold, significant correlations (*p* < 0.05)

### Multiple Regression Analyses

The parameters of the multiple regression models for the prediction of prosocial behavior by the independent variables are presented in Table 2. No single predictor was statistically significant in this analysis.

**Table 2:** Regression models estimating the prediction of prosocial behavior by attention, non-verbal intelligence and proactive (Model 1) and reactive aggression (Model 2)

Covariates	*b*	*SE*	*β*	*t*	*R*^2^	*F *(df)	*p*
*Model 1*							
					0.061	1.306 (3,63)	0.281
Proactive aggression	0.067	0.078	0.108	0,851			0.398
Sustained attention	0.011	0.006	0.214	1.618			0.111
Fluid intelligence	− 0.062	0.056	− 0.148	− 1.111			0.271
*Model 2*							
					0.050	1.054 (3,63)	0.375
Reactive aggression	− 0.004	0.055	− 0.010	− 0.077			0.939
Sustained attention	0.010	0.007	0.211	1.588			0.117
Fluid intelligence	− 0.069	0.055	− 0.166	− 1.250			0.216

### Moderation Analysis

#### Proactive Aggression

The parameters of the moderation model for the prediction of prosocial behavior by proactive aggression is presented in Table 3. The model was statistically significant (*R*^*2*^ = 0.2763, *F*(8,55) = 2.6244, *p* = 0.0165), as well as the interactions 1 (proactive aggression with attention; *p* = 0.0062) and 2 (proactive aggression with intelligence; *p* = 0.0055, Table 3). This means that attention and intelligence function as moderators in the prediction of prosocial behavior by proactive aggression. The effect of proactive aggression on prosocial behavior was significant (*p* = 0.0099), and intelligence accounted for the model beyond the contributions of the proactive aggression and the interactions (*p* = 0.0148). All other variables did not contribute significantly to the model.

**Table 3:** Regression coefficients for proactive aggression as an antecedent variable

Variable	*β*	Mean	SE	LLCI	ULCI
Constant	1.3696	0.8588	5.6183	− 12.2333	10.9699
Proactive aggression	**0.7665**	**0.7940**	**0.4058**	**0.0614**	**1.7199**
Attention	− 0.0599	− 0.0519	0.0305	− 0.1009	0.0097
Interaction 1	**0.0058**	**0.0053**	**0.0025**	**0.0001**	**0.0097**
Intelligence	**0.4318**	**0.4671**	**0.2756**	**0.0116**	**1.1138**
Interaction 2	− **0.0409**	− **0.0432**	**0.0212**	− **0.0930**	− **0.0075**
Age	− 0.1483	− 0.1230	0.4441	− 1.0391	0.7052
Sex	− 0.2821	− 0.3213	0.4416	− 1.1830	0.5453
Working memory	− 0.0344	− 0.0871	0.1385	− 0.4689	0.1033

Significant values are highlighted

*Interaction 1* proactive aggression and attention interaction, *Interaction 2* proactive aggression and intelligence interaction

The interaction between proactive aggression, attention, and intelligence significantly adds 19% proportion of variance explained to the model (*R*^2^ change = 0.19, *F*(2,47) = 6.2012, *p* = 0.0041). Therefore, the effect of proactive aggression in prosocial behavior was dependent (conditional) on both attention and intelligence.

Regarding conditional effects, levels of attention and intelligence had a different impact on prosocial behavior depending on the child’s proactive aggression levels, as seen in Table 4. The greater the proactive aggression scores, the lower the prosocial behavior among children with low levels of attention and high levels of intelligence (effect 3). This relationship between the behavioral variables was inverted when the child had medium–high levels of attention and low-medium levels of intelligence (effects 4, 5, 7, and 8). That is, at these levels, the higher the proactive aggression the greater the prosocial behavior.

**Table 4:** Conditional effects of proactive aggression in prosocial behavior among values of attention and intelligence

Attention	Effect	Intelligence	b	SE	*t*	*p*	95% CI	
							LL	UL
Low	1	Low	− 0.06	0.16	− 0.36	0.72	− 0.38	0.26
	2	Medium	− 0.26	0.15	− 1.80	0.08	− 0.55	0.03
	3	High	− **0.43**	**0.16**	− **2.72**	**0.01**	− **0.74**	− **0.11**
Medium	4	Low	**0.36**	**0.11**	**3.25**	**< 0.01**	**0.14**	**0.58**
	5	Medium	**0.16**	**0.08**	**2.07**	**0.04**	**0.01**	**0.31**
	6	High	− 0.01	0.09	− 0.08	0.91	− 0.18	0.17
High	7	Low	**0.44**	**0.12**	**3.64**	**< 0.01**	**0.20**	**0.69**
	8	Medium	**0.24**	**0.09**	**2.71**	**0.01**	**0.06**	**0.42**
	9	High	0.08	0.10	0.78	0.44	− 0.12	0.27

Significant values are in bold. Attention values: low = − 43, medium = 29, high = 43. Intelligence values: low = 14, medium = 19, high = 23

*SD* standard deviation, *Min* minimum, *Max* maximum. For correlations, in the bottom of the diagonal are the Pearson’s values and in the top the significance index. In bold, *p* < 0.05

**Fig. 2:**
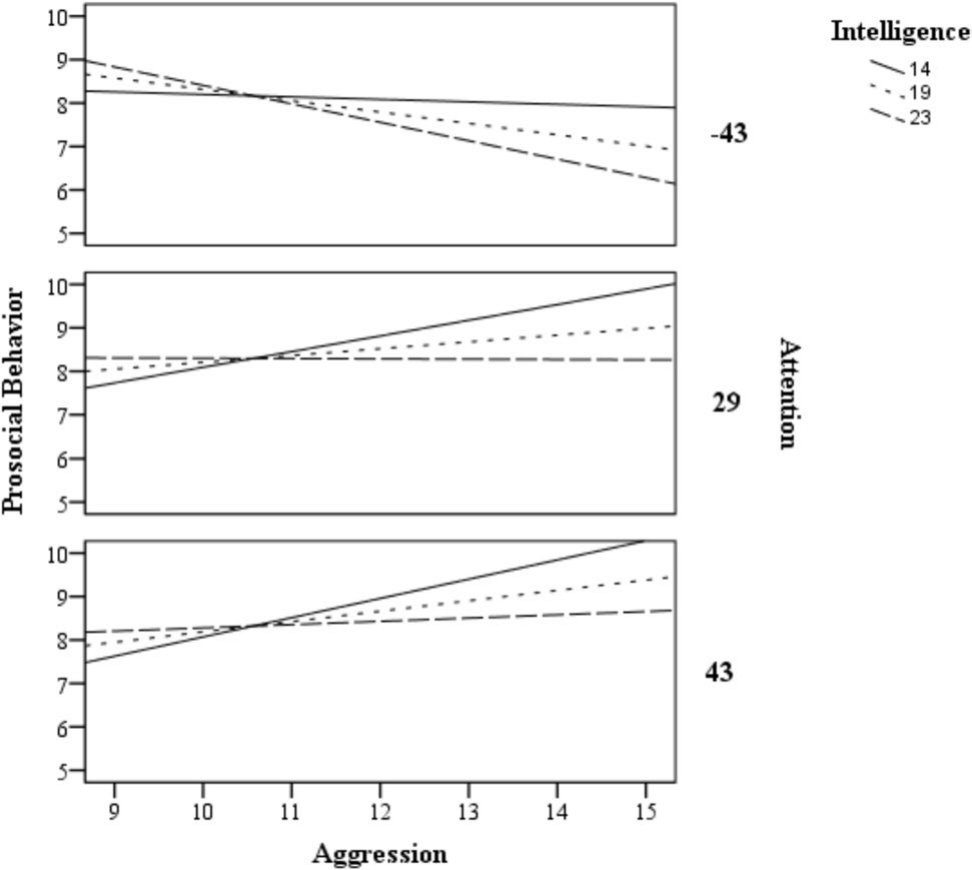
Conditional effects of proactive aggression on prosocial behavior at the levels of the moderators (16th, 50th and 84th percentiles)

These effects are graphically depicted in the Fig. 2. The graph is horizontally divided between levels of attention and the lines represent the intelligence levels. Note that, when the attention is low, there is a negative slope of the lines for any intelligence level, representing a decrease in prosocial behavior with an increase in peers’ aggression. This relation is opposite when the attention is medium or high, with positive slopes, representing an increase in prosocial behavior with an increase in proactive aggression, at least for the children classified at the low and medium intelligence levels. When intelligence is high, the prosocial behavior tends to be constant at the medium and high levels of attention at whatever proactive aggression levels.

#### Reactive Aggression

The multiple moderation model for the prediction of prosocial behavior by reactive aggression with attention and intelligence as moderators was not statistically significant (*R*^2^ = 0.2263, *F* (8,55) = 2.0110, *p* = 0.0620). Also, only the interaction between reactive aggression and attention was different from zero (Online Resource 2). This demonstrates that, in this model, attention with intelligence are not moderators of reactive aggression.

## Discussion

This study explored the prediction of prosocial behavior by direct aggression (proactive and reactive), and the role of attention and intelligence in relationships among children at risk of behavioral problems. Particularly, we had two aims: 1) to investigate the individual contribution of the independent variables in the prediction of prosocial behavior; 2) to investigate the conditional effect of cognitive processes (sustained attention and intelligence) in the prediction of prosocial behavior by aggression. Our results showed that the relationship between proactive aggression and prosocial behavior may be shadowed if we consider the variables isolated (correlations; multiple regression). Considering an additive multiple moderation, this relationship is conditioned by fluid intelligence and sustained attention (moderation analysis).

Previous studies that investigated the relationship between direct aggression and prosocial behavior usually found a negative (low to moderate) association among these variables [25, 29, 41–46]. Our results stand at odds with these studies and agrees with others who did not find such association [38–40]. In our sample, aggression tended to present medium to high prevalence among the sample (percentiles 50th and 84th in Table 1), while prosocial behavior tended to equally spread in the distribution. Our descriptive results contradict studies that evaluated the co-occurrence of both behaviors in terms of the joint trajectory of physical aggression and prosocial behavior [9, 23, 24, 40]. While these studies indicated a prevailing profile of moderate to low aggression and high prosociality, for our sample, joint prevalences of high aggression and moderate to high prosocial behavior constitute the prevailing profile (see Online Resource 3). It is noteworthy that our results are descriptive, while previous studies used latent class analysis (e.g., [24]) or other more advanced techniques of grouping level analyses (e.g., nonlinear Mixture of Curves (MOC) in [9]). A larger sample of children at risk for behavioral problems should be pursued in future studies.

While children at risk for behavioral problems are expected to exhibit high rates of aggression (proactive and reactive), our finding that they can also exhibit high rates of prosocial behavior is striking. This may be due to environmental variables that were not considered in the present work such as peer acceptance and tolerance towards violence. Studies that relate aggression, prosocial behavior, and peer acceptance usually conclude that prosocial behavior is a protective factor against aggression through peer acceptance (e.g., [29, 42]). In other words, children with high levels of aggression and low prosocial behavior usually have lower acceptance from their peers. Moreover, in social environments where children’s aggressive actions or reactions are acceptable, opportunities and justifications for the child to continue acting aggressively may rise [42]. This is likely to occur in a vulnerable environment such as the one from our sample, although this hypothesis is yet to be proved.

The moderation analysis suggested that when cognitive variables are considered, the relationship between proactive aggression and prosocial behavior is changed (Fig. 2; Table 3). When considered individually in the model, intelligence is a protective factor for prosocial behavior, as higher levels of non-verbal intelligence predict higher levels of prosociality. Conversely, we demonstrated that an increase in proactive aggression is associated with a decrease in prosocial behavior among children with low sustained attention, and high levels of intelligence (see Table 4). It is possible that low-attention children, despite having enough problem-solving ability to deal with the social environment, fail to receive and properly understand and operate the social cues for a given social situation. At least for children with attention deficit hyperactivity disorders, problems related to peer interaction and social skills are well documented (see Frederick and Olmi [76] for a review). Our study does not allow to explain the specific pathways that lead to the observed interaction. For example, Yu and Smith [77] suggested that early parent–child social interactions contribute to the development of sustained attention. Therefore, sustained attention may interfere, or be influenced by social interactions. Longitudinal studies seeking to investigate the trajectories of these variables should shed light on this issue.

Conversely, increases in proactive aggression are associated with increases in prosocial behavior among children with medium to high attention and low to medium intelligence levels. In this case, it is possible that a high attention level compensates the low cognitive ability, so that they can handle and select different strategies in social situations despite their lower levels of intelligence. Another hypothesis is that strategies of prosocial compensation of maladjusted behavior may be involved. For example, using the Prosocial Cyberball Game, van der Meulen et al. [78] showed that prosocial compensating behavior was associated with activity in posterior cingulate cortex (PCC) among children. This cerebral area is related to functions such as cognitive regulation and control, necessary for performance of cognitively demanding tasks which require attention [79].

Finally, it is important to highlight that although reactive aggression was prevalent in our sample (see Online Resource 3), its function did not predict prosocial behavior, nor was moderated by cognitive abilities. It is possible that the characteristics of the sample explain our results. A ceiling effect was observed in reactive aggression score, with 50% of the sample achieving the total score (18 points). In fact, direct aggression also presents a negative skewness distribution, but smoother (37.5% of the sample achieved the total score of 15). It is possible that sample variability was not enough to demonstrate effects for the reactive aggression model (i.e., the model explained 22% of the variance, against 27% of the direct aggression model). A larger sample could offer a wider variability of this measure. A sample enriched for behavioral problems may explain, in part, the high prevalence of aggression (direct and reactive). Moreover, in a social environment where children are prone to be exposed to aggressiveness, such as the vulnerable environment of our sample, children may behave aggressively due to vicarious learning [80].

Our study adds new insights about the interactions between problem solving (non-verbal intelligence) and cognitive attention with aggression and prosocial behavior among children at risk for behavioral problems. Like any study, some limitations should be highlighted. As noted, our sample was limited in terms of its size and variability. We used a small sample size due logistic purposes (i.e., the interruption of data collection cause by the COVID-19 pandemic). Furthermore, fluid intelligence was used as the only variable when measuring intelligence, and verbal skills were not examined. Nevertheless, measures of verbal abilities do not show significantly higher g-loadings to those of non-verbal abilities [81], and among children, verbal and non-verbal skills have been shown to be moderately correlated [82]. Moreover, our design limited the target sample to children at risk of behavioral problems. Therefore, the model should be tested in a larger, community-based sample for cross validation. Despite these limitations, the model with the selected variables explained 27% of the variance in prosocial behavior. Exploring these relationships in larger and heterogeneous sample is encouraged.

Our study has many strengths. To our knowledge, this is a first contribution to the literature on the relationship between aggression and prosocial behavior that investigated the role of attention and intelligence in a moderation model. It is also a first study to investigate two functions of aggression forms–proactive and reactive—in the relation with those cognitive abilities in a same sample. Our results may be interpreted in a clinical perspective. We suggest that interventions targeting children at risk for behavioral problems consider their cognitive abilities, particularly the low attention and high intelligence. For educational settings, future research should explore the implications of these results for academic achievement and social interactions.

## Summary

A﻿ggression and prosocial behavior are different means of responding to the social environment, although generally the latter is recognized as more adaptive ways to respond to social demands. Little attention has been given to the impact of aggression on the expression of prosocial behavior, although the arising of aggression precedes prosocial behavior in terms of its development. Moreover, albeit some complex steps may be involved to solve problems and made decisions in the context of social interactions, which could require the use of cognitive skills, intelligence and attention are hardly ever considered in the study of the influences of aggression and prosocial behaviors. Therefore, this study aimed to explore the influence of attention and intelligence in the prediction of prosocial behavior by direct aggression (proactive or reactive). The study was carried out with 64 school-aged children (6–8 years) screened for risk of behavioral problems by the Strengths and Difficulties Questionnaire. To this end, first, the prediction of prosocial behavior by direct aggression (proactive or reactive), attention, and intelligence were investigated by multiple regression models. Lastly, the conditional effect of attention and intelligence in the prediction of prosocial behavior by proactive and reactive aggression was analyzed by additive multiple moderation models. There was no association between the variables, and aggression (proactive or reactive), attention, and intelligence did not linearly predict prosocial behavior. Conversely, conditional effects were found, but only for the proactive aggression model: (1) negative impacts on prosocial behavior were observed among children with low attention and high intelligence performance; (2) medium and high levels of attention showed to be protective factors among low to medium intellectual ability children. These findings suggest that the relationship between proactive aggression and prosocial behavior may be shadowed if the conditionality of the cognitive variables is not considered. In the first case, it is possible that low-attention children, despite having enough problem-solving ability (intelligence) to deal with the social environment, fail to receive and properly understand and operate the social cues in the social interaction. Secondly, a high attention level compensates the low problem-solving ability, so that children can handle and select different strategies in social interactions despite their lower levels of intelligence. In a clinical perspective, interventions targeting children at risk for behavioral problems should consider their cognitive abilities, particularly the low attention and high intelligence.

## Supplementary Information

Below is the link to the electronic supplementary material.

Supplementary file1 (DOCX 14 KB)

Supplementary file2 (DOCX 14 KB)

Supplementary file3 (DOCX 16 KB)

## Data Availability

Data will be not available due ethical issues from Brazilian’s regulation. Syntaxes and outputs may be available upon request.

## Footnotes

1 Population size of the participants regions in 2010 were approximately: Centre (400,000); North (2,200,000); South (3,500,000); and East (4,000,000). Although the population of South region is more similar in size to East than North region, for logistic reasons we did not match them (because South population is dispersed in a very extensive and hazardous area) [63].

2 In the Brazilian version of the SDQ questionnaire, the cutoffs for difficulty are: 0–13 (normal); 14–16 (borderline); and ≥ 17 (abnormal). By the time of data collection, in the beginning of 2020, sample recruitment was challenging, because endorsement of the families was low, mainly in the experimental group. Also, most families interested in the study came from families whose children did not present behavioral problems at all. Our first cutoff was 14 for recruitment, then we changed to 10 based on previous studies [66],^67^ using a sample of families in vulnerability settings, as our target sample. Therefore, the sample is enriched for, but not limited by, children with behavioral problems.

3 In fact, two children of each group were evaluated and after removed because they aged 5 years old yet. Therefore, complete data collection is *n* = 66.
