# Supplementary material for: Relationship Between Direct Aggression and Prosocial Behavior: The Role of Attention and Intelligence Among Children at Risk for Behavioral Problems
Source: Child Psychiatry Hum Dev. 2024 Aug 16;57(3):771–82. doi: 10.1007/s10578-024-01738-7 (PMC13201365; doi:10.1007/s10578-024-01738-7)
Supplement: Supplementary file 1 — Supplementary file1 (DOCX 14 KB) [file 10578_2024_1738_MOESM1_ESM.docx]

**Online Resource 1.**

*Demographic characteristics of the Sample*

| Variables | Measure | *n* = 64 |
| --- | --- | --- |
| Age | Range | 6 – 8 |
|  | Mean (S.D.) | 6.50 (0.60) |
| Sex | % Female | 42.2 |
|  | % Male | 57.8 |
| Grade | % 1^st^ grade | 43.8 |
|  | % 2^nd^ grade | 56.3 |
| Socioeconomic classification | Range | B2 – D/E |
|  | Mode (Mean income) | C2^1^ (R$1.894,95)^2^ |
| Mother | % Have mother | 95.3 |
|  | % < High school | 26.2 |
|  | % ≥ High school | 73.8 |
| Father | % Have father | 90.6 |
|  | % < High school | 41.4 |
|  | % ≥ High school | 58.6 |

*Note*. ¹ Brazilian Criteria is divided into six strata and comes from A to D-E. For this criterion, C2 represents poor (but not extremely poor) class. ^²^ By the time of data collection, this represented almost U$450/month.
