# Supplementary material for: Relationship Between Direct Aggression and Prosocial Behavior: The Role of Attention and Intelligence Among Children at Risk for Behavioral Problems
Source: Child Psychiatry Hum Dev. 2024 Aug 16;57(3):771–82. doi: 10.1007/s10578-024-01738-7 (PMC13201365; doi:10.1007/s10578-024-01738-7)
Supplement: Supplementary file 2 — Supplementary file2 (DOCX 14 KB) [file 10578_2024_1738_MOESM2_ESM.docx]

**Online Resource 2.**

*Regression Coefficients for Reactive Aggression as an Antecedent Variable*

| Variable | β | Mean | SE | LLCI | ULCI |
| --- | --- | --- | --- | --- | --- |
| Constant | 6.4268 | 7.4731 | 5.6067 | -3.2871 | 19.1352 |
| Reactive aggression | 0.3351 | 0.2576 | 0.3287 | -0.4061 | 0.8670 |
| **Attention** | **-0.0581** | **-0.0570** | **0.0341** | **-0.1277** | **-0.0032** |
| **Int­eraction 1** | **0.0047** | **0.0046** | **0.0022** | **0.0007** | **0.0090** |
| Intelligence | 0.2668 | 0.1830 | 0.2558 | -0.3337 | 0.6511 |
| Interaction 2 | -0.0212 | -0.0179 | 0.0173 | -0.0504 | 0.0169 |
| Age | -0.2027 | -0.1695 | 0.4697 | -1,1447 | 0.7417 |
| Sex | 0.0667 | -0.0971 | 0.4697 | -1.0921 | 0.9045 |
| Working memory | -0.0406 | -0.0998 | 0.1367 | -0.4875 | 0.0372 |

*Note.* Significant values are highlighted. Confidence intervals generated with bootstrapping of 5000 bootstrap samples.

Interaction 1 = Reactive aggression and attention interaction

Interaction 2 = Reactive aggression and intelligence interaction
