# Supplementary material for: Relationship Between Direct Aggression and Prosocial Behavior: The Role of Attention and Intelligence Among Children at Risk for Behavioral Problems
Source: Child Psychiatry Hum Dev. 2024 Aug 16;57(3):771–82. doi: 10.1007/s10578-024-01738-7 (PMC13201365; doi:10.1007/s10578-024-01738-7)
Supplement: Supplementary file 3 — Supplementary file3 (DOCX 16 KB) [file 10578_2024_1738_MOESM3_ESM.docx]

**Online Resource 3.**

*Cross-Tabulation of Levels (Proportions) of Prosocial Behavior and Direct Aggression (Proactive and Reactive)*

|  |  | Prosocial Behavior | | |  | χ² |
| --- | --- | --- | --- | --- | --- | --- |
|  | Percentiles | 16^th^ | 50^th^ | 84^th^ | Total |  |
| Proactive Aggression | 16^th^ | **4.7%** | 7.8% | 6.3% | 18.8% | 2.770  *p* = 0.597 |
|  | 50^th^ | 15.6% | 12.5% | 15.6% | 43.8% |  |
|  | 84^th^ | 6.3% | 14.1% | **17.2%** | 37.5% |  |
|  | Total | 26.6% | 34.4% | 39.1% | 100% |  |
| Reactive Aggression | 16^th^ | **3.1%** | **3.1%** | 9.4% | 15.6% | 2.575  *p =* 0.631 |
|  | 50^th^ | 10.9% | 12.5% | 10.9% | 34.4% |  |
|  | 84^th^ | 12.5% | **18.8%** | **18.8%** | 50.0% |  |
|  | Total | 26.6% | 34.4% | 39.1% | 100% |  |

*Note.* df = 4.

In bold, higher and lower proportions of participants at the cross-tabulated percentiles.
